# Case report: Long-term efficacy and safety of semaglutide in the treatment of syndromic obesity in Prader Willi syndrome - case series and literature review

**DOI:** 10.3389/fendo.2024.1528457

**Published:** 2025-01-21

**Authors:** Andrijana Koceva, Katarina Mlekuš Kozamernik, Andrej Janež, Rok Herman, Simona Ferjan, Mojca Jensterle

**Affiliations:** ^1^ Department of Endocrinology and Diabetology, University Medical Center Maribor, Maribor, Slovenia; ^2^ Faculty of Medicine, University of Maribor, Maribor, Slovenia; ^3^ Department of Endocrinology, Diabetes and Metabolic Diseases, University Medical Center Ljubljana, Ljubljana, Slovenia; ^4^ Faculty of Medicine, University of Ljubljana, Ljubljana, Slovenia

**Keywords:** Prader-Willi syndrome, obesity, GLP-1 receptor agonist, semaglutide, metabolic surgery

## Abstract

**Introduction:**

Prader-Willi syndrome (PWS) is the most prevalent cause of syndromic obesity. Obesity development in PWS is driven by dysfunction in neural pathways involved in satiety and reward, dysregulation in hormones regulating satiety and food intake, altered body composition and reduced energy expenditure, as well as the presence of various hormone deficiencies. As hyperphagia, satiety dysfunction and consequent food-seeking behaviors are intrinsic to PWS, obesity management can be challenging.

**Case series:**

We present a long-term follow-up of treatment with GLP-1 receptor agonist (GLP-1 RA) semaglutide in three patients with PWS without diabetes, one of whom had previously undergone metabolic surgery. Semaglutide treatment at dosages from 0.5 mg to 2 mg weekly demonstrated variable efficacy, from preventing further weight gain in patient 1, to achieving weight loss of up to 14.4% and 11% relative to baseline, in Patient 2 and Patient 3. It was well tolerated, even after metabolic surgery.

**Conclusion:**

Long-term randomized placebo-controlled trials with larger sample sizes are needed to provide stronger evidence on the long-term efficacy and safety of semaglutide for obesity treatment in PWS as well as explore the potential synergistic effects of GLP-1 RA treatment combined with other therapeutic interventions.

## 1 Introduction

Prader-Willi syndrome (PWS) is a rare genetic disorder characterized by impaired hypothalamic development and function with an estimated incidence of 1 per 20,000 newborns (1). It represents the most common cause of syndromic obesity and obesity is present in 40% of children and 82-98% of adults with PWS (2). Patients with PWS have reduced life expectancy with an estimated 3% annual mortality rate compared to approximately 1% in the general population, with obesity, hyperphagia and food-related behavior being important risk factors for increased mortality (3–6).

Patients with PWS experience complex nutritional, neurodevelopmental, metabolic and behavioral changes that drive obesity development (1). Hypothalamic dysfunction and altered hypothalamic structure impair satiety response, with delayed or even absent activation of satiety centers following glucose or food intake (7–10). Additionally, hyperactivation in the subcortical reward pathways and hypoactivation in the cortical inhibitory regions after eating demonstrate heightened food motivation and reduced self-regulation (11–13). Altered body composition with increased fat mass and decreased muscle mass is accompanied by a 20-46% reduction in total energy expenditure, including reduced resting, sleeping and activity energy expenditure (14, 15). Growth hormone deficiency, hypothyroidism and hypogonadism commonly found in patients with PWS, also contribute to an increased fat-to-lean mass ratio and reduced energy expenditure, both of which ultimately promote weight gain (2, 16). Lastly, increased levels of some orexigenic hormones, such as ghrelin and decreased levels of anorexigenic hormones also have a role in the development of obesity (17–22).

Obesity management in PWS typically includes a calorie-restricted diet, strict control of food access, regular physical activity and behavioral interventions (23). Although there is no approved pharmacological treatment, various anti-obesity medications have been investigated in PWS, including GLP-1 RAs. The weight-reducing effects of GLP-1 RAs are predominantly mediated through multiple central mechanisms targeting satiety and reward pathways. GLP-1 RAs activate GLP-1 receptors in the nucleus accumbens and the ventral tegmental area, which represent a key reward pathway, as well as modulate energy intake and expenditure by activating anorexigenic proopiomelanocortin (POMC)/cocaine and amphetamine regulated transcript (CART) neurons and inhibiting orexigenic neuropeptide Y (NPY)/agouti-related peptide (AgRP) neurons in the arcuate nucleus (24–27). Additionally, GLP-1 RAs act on neuronal GLP-1 receptors in the nucleus of the solitary tract and circumventricular organs, activating regions such as the central amygdala, bed nucleus of the stria terminalis, parabrachial nucleus and paraventricular nucleus, which are also involved in regulating energy intake and feeding behavior (25, 28). Since PWS is characterized by impaired hypothalamic regulation and neurodevelopmental anomalies in the satiety and reward regulating pathways, the question arises of whether GLP-1 RAs are effective and consistent in this population. To our knowledge, the long-term effectiveness and safety of semaglutide treatment in patients with PWS without diabetes, especially following previous metabolic surgery, has not yet been documented in the literature.

## 2 Case series

### 2.1 Patient 1

A 28-year-old female with PWS, resulting from uniparental disomy, was evaluated at our Endocrinology Unit. Her medical history included hypogonadism, growth hormone deficiency, asymptomatic cholecystolithiasis (gallbladder sludge) and obesity. During her initial consultation, her weight was 109 kg, her height was 146.5 cm, and her BMI was 50.8 kg/m^2^. She was initially treated with a calorie-restricted diet and subsequently admitted to a specialized “group home center”, however, her weight continued to progressively increase. She did not have diabetes mellitus, her HbA1c was 5.8%. On follow-up, she weighed 118 kg, had a waist circumference (WC) of 128 cm and her waist-to-hip ratio (WHR) was 0.98. She agreed on growth hormone supplementation with somatropin (0.2 - 0.4 mg daily) and GLP-1 RA treatment with semaglutide. Semaglutide was commenced at a dose of 0.25 mg per week for the first four weeks, increased to 0.5 mg per week for the next month, and then further increased to 1 mg per week. Semaglutide treatment over four months led to a weight reduction of 5 kg, from 118 kg (BMI 55 kg/m^2^) to 113 kg (BMI 52.7 kg/m^2^). Although appetite and satiety were not assessed formally, the patient reported appetite suppression as well as better physical performance. After discontinuing treatment due to the temporary unavailability of the drug, she regained 7 kg in three months, increasing her body weight to 120 kg (body mass index 59.9 kg/m2). Upon the next follow-up, semaglutide was re-introduced for the next 33 months, which led to a 7 kg weight reduction during the first two years of treatment (113 kg, BMI 52.65 kg/m^2^), followed by a slight weight regain during the third year. Nonetheless, semaglutide facilitated maintaining a stable weight of 117 kg (BMI 54.5 kg/m^2^, WC 128 cm, WHR 0.96), without further weight regain and her HbA1c remained stable at 5.8%. Treatment was well tolerated without reported side effects.

### 2.2 Patient 2

A 39-year-old female with PWS resulting from mosaic maternal uniparental disomy was evaluated at our Endocrinology Unit. Her medical history included growth hormone deficiency, osteoarthrosis and morbid obesity for which she underwent metabolic surgery at the age of 29, first a gastric band surgery and subsequently Roux-en-Y gastric bypass. Despite an initial weight loss of approximately 50 kg, she ultimately regained most of her lost weight. During our follow-up she reached her maximum weight of 174 kg, her height was 145 cm, and her BMI was 82.8 kg/m^2^. She had a WC of 155 cm and her WHR was 0.99. Her HbA1c was 6% and her fasting glucose was 5.3 mmol/l. After discussing potential treatment options, the patient agreed to pharmacological treatment. She started GLP-1 RA treatment with semaglutide, and after two months growth hormone replacement with somatropin (0.2 - 0.4 mg daily) was also initiated. Semaglutide was initiated at a dose of 0.25 mg weekly for the initial month and elevated to 0.5 mg weekly. During initial dose escalation, transient dyspepsia and regurgitation occurred, out of caution semaglutide treatment was continued at a lower dose of 0.5 mg weekly for the following five months, which led to a weight reduction of 8 kg. The patient reported appetite suppression immediately after starting semaglutide treatment. Following growth hormone initiation, she reported ability to prolong the duration of daily activities and that she experienced improved physical function and increased muscle strength. Appetite suppression and muscle function were not assessed formally. Due to shortage, a two-month pause in treatment occurred without weight gain, after which semaglutide was re-introduced and gradually increased to 1 mg over the next four months. This led to an additional 6 kg weight loss (weight 160 kg, BMI 76.1 kg/m^2^) without reported side effects. This was followed by another temporary cessation of semaglutide treatment due to unavailability of the drug. Following GLP-1 RA discontinuation, the patient experienced an increase in appetite, regained 7 kg (weight 167 kg, 79.4 kg/m^2^) and was also later diagnosed with type 2 diabetes. She was started on a slow-release metformin, and at a later stage, semaglutide was re-introduced. The patient reported dysphagia and belching, which were also present even before the initiation of semaglutide treatment, and these symptoms resolved spontaneously in the following months. Semaglutide treatment at a dose of 1 mg weekly resulted in an 11 kg weight reduction over nine months, followed by an additional 7 kg weight loss over the next eleven months, once the dosage was elevated to 2 mg weekly. Her last recorded weight was 149 kg (BMI 70.9 kg/m^2^), with WC of 140 cm and WHR of 0.96.

### 2.3 Patient 3

A 25-year-old male with PWS, resulting from a uniparental maternal disomy was seen at our Endocrinology Unit. His medical history included hypogonadism, growth hormone deficiency, obesity and obstructive sleep apnoea treated with CPAP. He was receiving testosterone replacement, but his parents refused growth hormone supplementation during childhood. Before follow-up, his weight was 103 kg, height 158 cm and body mass index (BMI) 41.3 kg/m^2^. Due to progressive weight gain (137 kg, BMI 54.9 kg/m^2^, WC 140 cm and WHR 1.0.) despite non-pharmacological interventions with the support of a dietician, kinesiologist and psychologist, treatment with semaglutide was introduced. Semaglutide was initiated at a dose of 0.25 mg weekly for the first month, 0.5 mg weekly during the second month, and then increased to 1 mg weekly. Although not assessed in a formal manner, he reported appetite suppression and achieved a weight loss of 15 kg (122 kg, BMI 48.9 kg/m^2^, WC of 134 cm, WHR 0.97) over the first eight months of treatment and maintained during the next three months. His glycaemic control remained stable with an HbA1c of 5.8%. Semaglutide treatment was well tolerated without reported side effects.

**Figure 1:**
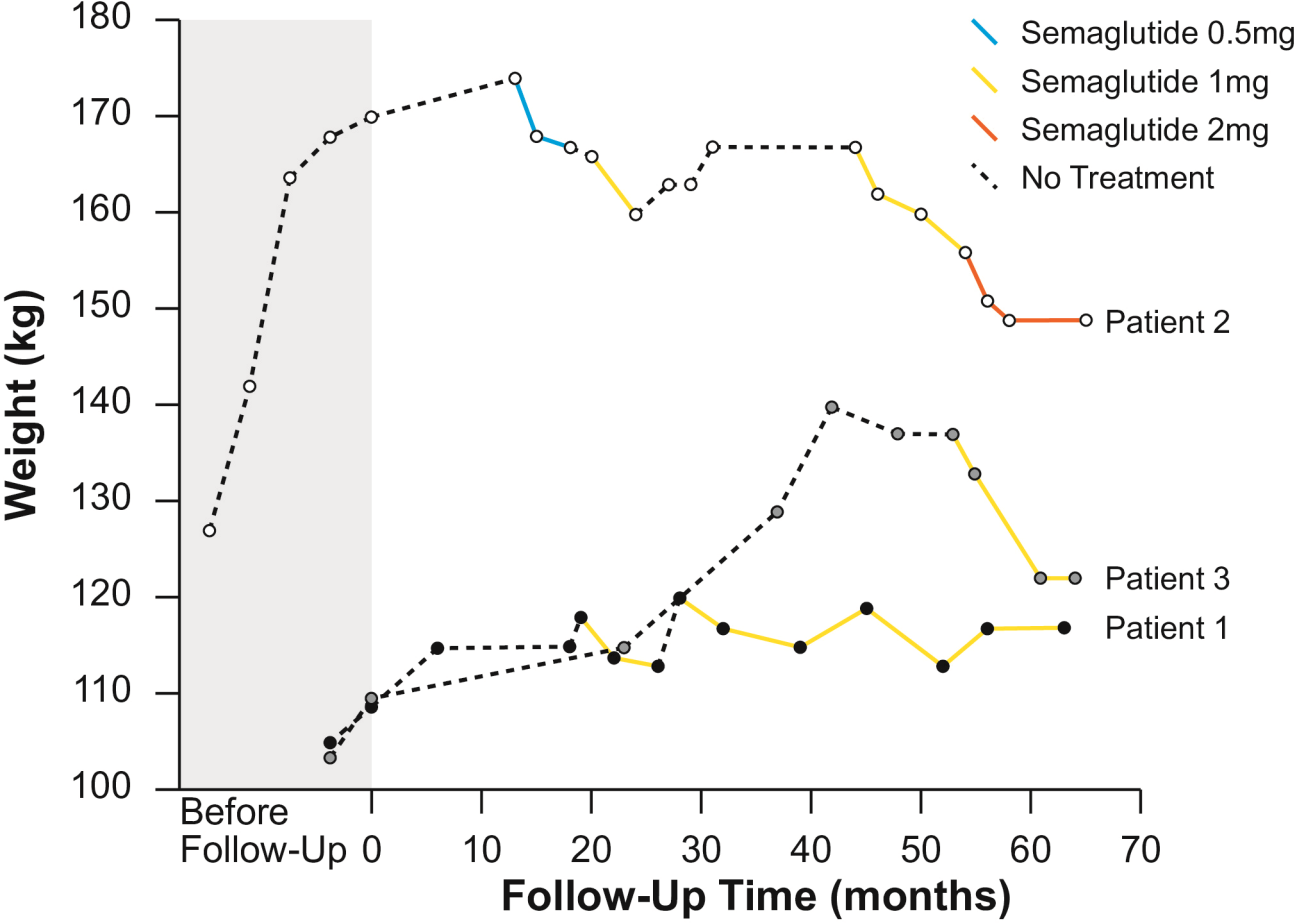
Weight change over time for patients 1, 2 and 3.

**Table 1** summarizes patient characteristics, treatment details and weight loss outcomes, while **Figure 1** shows the trajectory of the patient’s weight change before and after the introduction of GLP-1 RA treatment with semaglutide.

**Table 1:** Patient characteristics, treatment details and key outcomes.

Patient	Patient 1	Patient 2	Patient 3
Sex	Female	Female	Male
Age	28	39	25
Growth hormone treatment	Yes	Yes	No
HbA1c (%) (at baseline)	5.8%	6%	6%
Weight (kg) (at baseline)	118 kg	174 kg	137 kg
BMI (kg/m^2^) (at baseline)	55 kg/m^2^	82.8 kg/m^2^	54.9 kg/m^2^
Highest semaglutide dose	1 mg	2 mg	1 mg
Total semaglutide treatment time (months)	37 months	30 months	11 months
Weight (kg) (at last follow-up)	117 kg	149 kg	122 kg
BMI (kg/m^2^) (at last follow-up)	54.5 kg/m^2^	70.9 kg/m^2^	48.9 kg/m^2^
Weight loss (%) (relative to baseline)	- 0.85%	- 14.4%	- 11%

## 3 Discussion

GLP-1 RAs may provide therapeutic benefits for managing obesity in PWS through several mechanisms. These include anorectic effects driven by previously discussed central modulation of satiety and reward pathways and peripheral activation of vagal afferents, delayed gastric emptying, anti-inflammatory properties, potential impact on energy expenditure and the ability to reduce ghrelin levels, which are inherently elevated in PWS (23–26, 28). The use of various GLP-1 RAs in PWS has been extensively reported in the literature, mostly in patients with concomitant diabetes. Liraglutide at a daily dose of 3 mg is the first GLP-1 RA registered for non-syndromic obesity treatment, with a mean weight change of 8% in individuals with obesity without diabetes (29) and 6% in individuals with obesity and diabetes (30). Treatment with liraglutide at variable doses (0.9 mg to 1.8 mg daily) has been reported in case reports of patients with PWS and concomitant diabetes, showing heterogenous efficacy in BMI reduction from 1.3 kg/m^2^ to 11.3 kg/m^2^ (31–35), waist circumference reduction from 7 to 8 cm (32, 35), as well as HbA1c reduction from 1.9% to 6.5% (31–34). GLP-1 RA treatment in these reports was well tolerated, without reported adverse events, mostly combined with metformin (32–35) and/or empagliflozin (33, 34). In a systematic review of ten studies, Ng et al. analyzed GLP-1 RAs efficacy in 23 patients with PWS, sixteen (70%) of which were also diagnosed with diabetes. Patients were treated with exenatide (14/23) or liraglutide (9/23) and GLP-1 RAs treatment was again connected to a wide range of weight loss, from 1.5 to 16 kg/m^2^ in 10/14 cases, and an HbA1c improvement in 19/23 cases, without reported serious side effects (23). Although these studies reported favorable outcomes, others reported comparatively modest outcomes on weight control. In a review by Fintini et al., six patients with PWS and concomitant diabetes were treated with GLP-1 RAs, liraglutide (1.2 – 1.8 mg daily) in four patients and exenatide (20 mcg daily) in two patients, showing only a tendency for BMI and waist circumference reduction after 24 months of treatment (36). Another retrospective analysis of real-world data by Nolan et al. also reported that treatment with liraglutide (0.6 – 3 mg daily) in seven patients with PWS (in combination with topiramate in 3 individuals), resulted in a variable response with a median weight loss of 9 kg over 96 weeks (37). A recent multicentre placebo-controlled trial of liraglutide efficacy in adolescents and children with PWS without diabetes showed that although adolescents treated with liraglutide 3 mg had lower hyperphagia scores at week 52, changes in BMI standard deviation score from baseline to week 16 and week 52 did not significantly differ (38).

Semaglutide at a dose of 2.4 mg weekly is the first weekly long-acting GLP-1 RA approved for non-syndromic obesity treatment with a mean weight change of 14.9% in individuals with obesity without diabetes (39) and 9.6% in individuals with obesity and concomitant diabetes (40). Apart from greater average weight loss compared to liraglutide, once-weekly application may lead to improved treatment adherence in PWS. There are only a few published cases of semaglutide use in PWS, usually in the setting of concomitant diabetes using doses of 0.5 and 1 mg weekly, with limited evidence on the long-term efficacy and safety of semaglutide in PWS without diabetes. In a case report by Sani et al., the use of semaglutide 1 mg weekly over 12 months in a male with PWS and diabetes resulted in an HbA1c decrease of 3.9% and weight loss of 5.2 kg (41). Gimenez-Palos et al. assessed the impact of semaglutide treatment (0.5 – 1 mg weekly) over 12 to 36 months on weight reduction and glycemic control in four adults with PWS with concomitant diabetes, all of whom also started growth hormone replacement approximately 3 to 5 years before semaglutide initiation. Two of the four patients experienced an improvement in weight and glycaemic control; one patient had an initial improvement in both glycemia and weight followed by weight regain and deterioration of glycaemic control, and one patient experienced only glycemia improvement with weight gain (42).

Our case series presents the long-term efficacy and safety of semaglutide treatment in patients with PWS without diabetes as well as semaglutide efficacy and safety in PWS previously treated with metabolic surgery. Aligned with earlier findings, semaglutide treatment at dosages from 0.5 mg to 2 mg weekly demonstrated variable efficacy, from merely preventing further weight gain in Patient 1 to achieving weight loss of up to 14.4% and 11% relative to baseline, in Patient 2 and Patient 3, respectively. All patients reported appetite suppression and increased satiety during semaglutide treatment, which was diminished during treatment interruptions. Similarly, as seen in non-syndromic obesity (43, 44), semaglutide treatment discontinuation, which occurred in Patient 1 and Patient 2, resulted in weight regain. Mild transient gastrointestinal (GI)-related side effects were reported during dose escalation in the case of Patient 2, who had a history of metabolic surgery and no serious adverse events were reported during the observation period.

Type 2 diabetes mellitus is a common obesity-related complication in PWS and is diagnosed in 7-24% of adults with PWS compared to 5-7% in the general population (45, 46). In our case series, Patient 2 developed diabetes following weight regain during the temporary GLP-1 RA treatment discontinuation and semaglutide was successful in weight control before and after the onset of diabetes. Patient 1 and Patient 3 did not develop diabetes over the time of observation.

Growth hormone deficiency is diagnosed in between 40 and 100% of patients with PWS. There is increasing evidence of the benefits of growth hormone treatment in adults with PWS, such as a continued increase in lean mass and decrease in fat mass, improved muscle strength, improved mental and cognitive function and possibly improved quality of life (47–52). In our case series, Patients 1 and 2 started growth hormone replacement in adulthood, when obesity was concurrently addressed with semaglutide. We monitored the potential adverse effects of growth hormone replacement on fluid retention, blood pressure, glycemia and respiratory issues. Over the following months, both patients reported improved functional status, easier walking, prolonged ability for daily physical activities and increased muscle strength. However, as weight loss is also associated with enhanced physical fitness, these observed effects may not be attributed solely to growth hormone treatment.

The role of metabolic surgery is well-established in non-syndromic obesity, but its efficacy in PWS is controversial (53, 54). Studies show that despite initial weight reduction, patients with PWS inevitably experience weight regain and long-term efficacy is questionable (54–56). Patient 2 underwent two types of metabolic surgery, first gastric band and later Roux-en-Y gastric bypass, nevertheless she regained almost all lost weight. The efficacy of GLP-1 RAs, semaglutide and liraglutide, for the management of weight regain after bariatric surgery was documented by Jensen et al. in a retrospective observational study where they showed that two-thirds of weight regain following bariatric surgery can be lost with GLP-1 RAs treatment with only mild, transient and mostly GI-related adverse events reported in one-third of patients (57). Similarly, in our case series, Patient 2 experienced satisfactory weight loss during semaglutide treatment with only mild, transient, GI-related side effects during the initial dose escalation.

Our case series has several limitations. Although all patients reported appetite suppression and improvement in satiety, appetite was not objectively measured using the visual analog scale (58). Additionally, two patients started growth hormone treatment closely with the initiation of semaglutide, complicating the interpretation of the respective contributions of weight loss and growth hormone treatment on improvements in physical performance and fitness. Furthermore, we did not assess body composition changes systematically over the course of GLP-1 RA treatment due to patients’ reluctance. We continuously educated patients and caregivers about the importance of physical activity and a balanced diet with sufficient protein and fiber intake to maintain muscle mass. We also advised hydration and monitored vitamin D and other vitamins and minerals supplementation, especially in a patient after metabolic surgery. Moreover, in our country, semaglutide is not reimbursed for the treatment of obesity by the public health system, creating financial challenges for some patients. Additionally, intermittent shortages of semaglutide have made consistent access to the medication difficult. These factors collectively led to variations in semaglutide dosing and temporary interruptions in treatment. Lastly, the observational design, lack of standardized outcome measures, small sample size and heterogeneity of responses to semaglutide treatment, likely due to the complex nature of obesity in PWS, reduces the generalizability of the findings to a wider population.

Nonetheless, our case series also has notable strengths. It provides long-term follow-up of semaglutide treatment at different doses in PWS patients without diabetes, ranging from 11 months to 37 months. We have also provided insights into patients’ weight trajectories for a long period before the introduction of anti-obesity pharmacotherapy. Moreover, the case series included the follow-up of combined growth hormone and semaglutide treatment in two of the three patients. Our extended follow-up additionally provided insights into the effects of semaglutide treatment discontinuation in PWS. Finally, it is the first report documenting the favorable efficacy and safety of semaglutide use at three different doses (0.5 mg, 1 mg and 2 mg) in a patient with PWS without diabetes who had previously undergone metabolic surgery.

Future research should prioritize long-term randomized placebo-controlled trials with larger sample sizes to provide stronger evidence on the long-term efficacy and safety of semaglutide for obesity treatment in PWS as well as explore the potential synergistic effects of GLP-1 RA treatment combined with other therapeutic interventions. Additionally, studies should include objective measures of satiety and appetite, such as validated questionnaires, to assess the effectiveness of semaglutide in modulating food-seeking behavior. Furthermore, body composition changes, including fat and lean mass alterations, should be systematically assessed using standardized techniques to better understand semaglutide’s impact on metabolic health in this population.

## 4 Conclusion

In conclusion, due to its multifaceted and heterogeneous nature, a personalized approach is necessary for obesity management, especially in syndromic obesity like PWS where the underlying pathophysiology remains largely unknown. Semaglutide treatment in our case series of patients with PWS without diabetes led to weight reduction and/or weight maintenance, as well as to appetite suppression as reported by patients and caregivers. The treatment was safe without moderate or severe side effects. Due to the study’s design, small sample size, variation in dosing and variable results, the findings of this study are preliminary and highlight the need for further research.

## Data Availability

The original contributions presented in the study are included in the article/supplementary material. Further inquiries can be directed to the corresponding author.
